# Expanding the importance of HMERF titinopathy: new mutations and clinical aspects

**DOI:** 10.1007/s00415-019-09187-2

**Published:** 2019-01-21

**Authors:** Johanna Palmio, Sarah Leonard-Louis, Sabrina Sacconi, Marco Savarese, Sini Penttilä, Anna-Lena Semmler, Wolfram Kress, Tahseen Mozaffar, Tim Lai, Tanya Stojkovic, Andres Berardo, Ricardo Reisin, Shahram Attarian, Andoni Urtizberea, Ana Maria Cobo, Lorenzo Maggi, Sergei Kurbatov, Sergei Nikitin, José C. Milisenda, Farzad Fatehi, Monika Raimondi, Fernando Silveira, Peter Hackman, Kristl G. Claeys, Bjarne Udd

**Affiliations:** 1 Department of Neurology, Neuromuscular Research Center, Tampere University Hospital and University of Tampere, 33014 Tampere, Finland; 2 Institute of Myology, National Reference Center for Neuromuscular Disorders, University Hospital of Salpêtrière, UPMC, Paris, France; 3 Nice University Hospital, Université Côte d’Azur, Nice, France; 4 Folkhälsan Institute of Genetics and Medicum, Haartman Institute, University of Helsinki, Helsinki, Finland; 5 Department of Neurology, RWTH Aachen University, Aachen, Germany; 6 Institute of Neuropathology, RWTH Aachen University, Aachen, Germany; 7 Institute of Human Genetics, University of Würzburg, Würzburg, Germany; 8 Neurology Department, University of California, Irvine, Orange, CA USA; 9 Center of Research in Myology, UPMC Univ Paris, INSERM UMRS, Institut de Myologie, Sorbonne Universités, Paris, France; 10 Neuromuscular Unit, British Hospital, Buenos Aires, Argentina; 11 Reference Center for Neuromuscular Disorders and ALS, CHU La Timone 1338, Marseille, France; 12 Centre de Compétences Maladies Neuromusculaires Hendaye, Hendaye, France; 13 Neuroimmunology and Neuromuscular Diseases Unit, Foundation IRCCS Neurological Institute Carlo Besta, Milan, Italy; 14 Regional Medical Diagnostic Centre, Voronezh, Russia; 15 Regional Non-governmental Organization «Society of Neuro-Muscular Diseases Specialists», Moscow, Russia; 16 Muscle Research Unit, Internal Medicine Service, Hospital Clínic de Barcelona and CIBERER, Barcelona, Spain; 17 Iranian Center of Neurological Research, Neuroscience Institute, Tehran University of Medical Sciences, Tehran, Iran; 18 Clinica Moncucco, Via Moncucco 10, 6900 Lugano, Switzerland; 19 Hospital São Joao Porto, Porto, Portugal; 20 Department of Neurology, University Hospitals Leuven, Leuven, Belgium; 21 Laboratory for Muscle Diseases and Neuropathies, Department of Neurosciences, KU Leuven, Leuven, Belgium; 22 Department of Neurology, Vaasa Central Hospital, Vaasa, Finland

**Keywords:** Hereditary myopathy, Respiratory failure, Titin, Titinopathy, mutations

## Abstract

**Objective:**

Hereditary myopathy with early respiratory failure (HMERF) is caused by titin A-band mutations in exon 344 and considered quite rare. Respiratory insufficiency is an early symptom. A collection of families and patients with muscle disease suggestive of HMERF was clinically and genetically studied.

**Methods:**

Altogether 12 new families with 19 affected patients and diverse nationalities were studied. Most of the patients were investigated using targeted next-generation sequencing; Sanger sequencing was applied in some of the patients and available family members. Histological data and muscle MRI findings were evaluated.

**Results:**

Three families had several family members studied while the rest were single patients. Most patients had distal and proximal muscle weakness together with respiratory insufficiency. Five heterozygous TTN A-band mutations were identified of which two were novel. Also with the novel mutations the muscle pathology and imaging findings were compatible with the previous reports of HMERF.

**Conclusions:**

Our collection of 12 new families expands mutational spectrum with two new mutations identified. HMERF is not that rare and can be found worldwide, but maybe underdiagnosed. Diagnostic process seems to be complex as this study shows with mostly single patients without clear dominant family history.

## Introduction

Hereditary myopathy with early respiratory failure (HMERF, OMIM #603689) is characterized by proximal and/or distal muscle weakness, and early and severe diaphragmatic insufficiency [1–5]. In HMERF, respiratory failure can be a presenting symptom in an ambulant adult patient, which is not a common feature in other genetic myopathies [5–7]. Typical MRI pattern has been reported with fatty degeneration of semitendinosus and obturator muscles and anterolateral compartment of lower legs early in the disease course [4, 5, 8, 9, 10]. Muscle histopathology shows cytoplasmic bodies usually in subsarcolemmal necklace-like formation, occasional rimmed vacuoles and myofibrillar disorganization responsible for Z-disc alterations [11].

Titin gene mutations in exon 344 encoding the fibronectin-3 (FN3) domain in the A-band region of titin are associated with HMERF [4, 5]. The reported mutations mainly show an autosomal dominant inheritance pattern, and are usually private mutations except for the most frequently identified mutation in HMERF patients, c.95134T>C p.C31712R, found in more than 20 families in Europe and Asia (Table 1) [11–14].

**Table 1:** Dominant mutations in TTN A-band-exon 344 with clinical findings

Mutation	Nationality	No. of families (patients)	Distribution of common muscle weakness	Respiratory involvement	Other features (no. of patients)	References
c.95126C>A p.P31709H	Filipino-Caucasian	1 (1)	Proximal and distal lower limb weakness	1/1	–	^a^
c.95126C>G p.P31709R	French	1 (3)	Proximal, axial	2/3	–	[9]
c.95134T>C p.C31712R	British, Swedish, Spain/Canada, Finnish, Italian, Argentinian, Japanese, Chinese Afghan^a^, Russian^a^	33 (96)	Proximal, axial and/or distal myopathy neck flexion, ankle dorsiflexion, trunk, pelvic muscles	69/96	Calf hypertrophy (12), finger flexion/extension, scapular winging (6), contractures (3), rigid spine/kyphoscoliosis (5), dysphagia needing PEG (1), head drop (1), myalgia, cramps (1)	[4, 5, 9, 11, 13, 14, 15]^a^
c.95135G>A p.C31712Y	Japanese	1 (1)	Proximal LL	1/1^b^	–	[11]
c.95185T>C p.W31729R	German	1 (2)	Proximal and distal, neck flexion	2/2	Mild facial muscle weakness	[9]
c.95186G>T p.W31729L	Japanese	1 (20)	Ankle dorsiflexion, finger extension	7 (not all examined)	Dysphagia and dysarthria	[10]
c.95187G>C p.W31729C	British, Portuguese	4 (4)	Distal weakness	4/4	Mild kyphosis (1), scapular winging (1)	[9, 16]^a^
c.95346_95354del p.R31783_V31785del	Japanese	1 (1)	Distal LL, UL weakness	1/1^b^	Myalgia	[11]
c.95351C>T p.A31784V	French, Argentinian	3 (6)	Axial, proximal and distal, neck flexors, ankle dorsiflexion	6/6	Scapular winging (2), dysphonia (2), calf hypertrophy (1)	^a^
c.95358C>G p.N31786K	Brazilian, Iranian^a^	1 (1)	Proximal UL, LL, ankle dorsiflexion	0/1	Scapular winging	[15]^a^
c.95371G>C p.G31791R	Japanese	1 (1)	Fatigability	1/1	–	[11]
c.95372G>A p.G31791D	North American, Japanese	2 (6)	Proximal LL, distal LL, neck muscles, UL	2/5 + 1/1^b^	Calf hypertrophy (4), head drop	[11, 14]
c.95372G>T p.G31791V	Japanese	1 (1)	Distal LL	1/1^b^	–	[11]

*PEG* percutaneous endoscopic gastrostomy tube feeding, *LL* lower limbs, *UL* upper limbs

^a^In the present study

^b^Asymptomatic, found on pulmonary function tests

We describe here clinical features, pulmonary function tests, histopathological and muscle MRI findings of 19 HMERF patients from 12 families and diverse ethnic origins. Five heterozygous *TTN* A-band mutations were identified of which two are previously unreported.

## Methods

### Patients

**Fig. 1:**
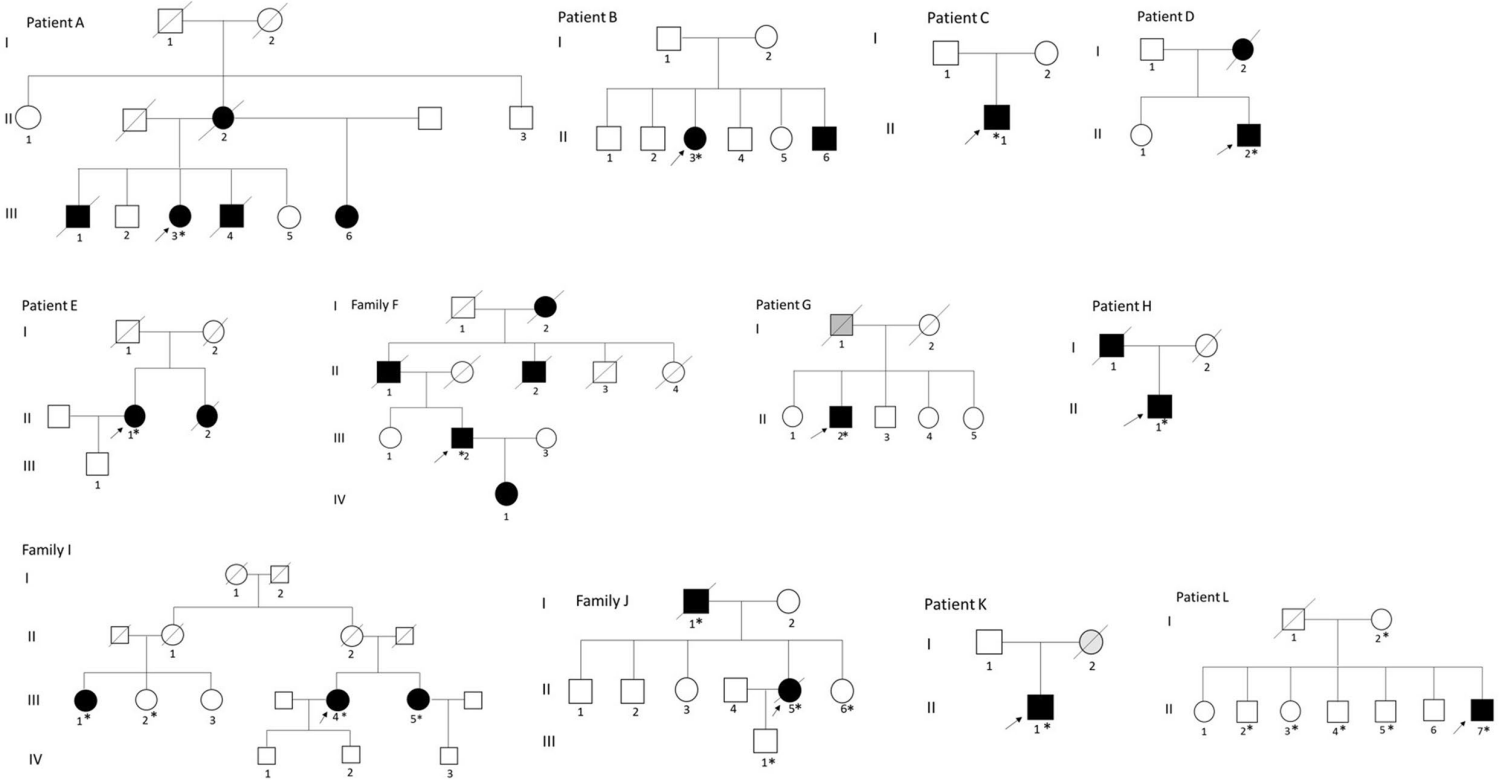
Pedigree of the families. DNA was collected from individuals marked with an asterisk*. Filled symbols are affected and open symbols unaffected family members. Grey symbols are family members that are possibly affected

The patients belonged to 12 unrelated families (Fig. 1): one Filipino/Caucasian (A), one Afghan (B), two Italian (C, D), one Spanish (E) patient, one Russian family (F), two Portuguese patients (G, H), two French families (I, J), one Argentinian (K), one Iranian (L) patient. Family I was from the East of France, with two sisters and her German cousin affected, while family J was from the South of France with two members affected. In the Russian family F father and daughter were examined and similarly affected. There were no other family members diagnosed with specific muscle disease in the rest of the families, although, some patients had relatives with a history of muscle weakness and/or respiratory problems (Fig. 1/Table 2). Those family members were already deceased or otherwise not available for this study.

**Table 2:** Clinical and genetic data of HMERF patients

TTN A-band-exon 344 c.95126C>A, p.P31709H (novel mutation)							
Filipino–Caucasian patient A							
Patient	Sex/age	Age at onset	First symptoms	Muscle weakness findings at examination	Respiratory symptoms	CK/IU/L	Biopsy
A: III-3	F/57	31	Proximal and distal lower limb weakness	Neck flexors, hip flexors, ankle dorsiflexors, abductor digiti minimi	FVC 43%, NIV	Normal	RV, myofibrillar aggregates
TTN A-band-exon 344 c.95134T>C, p.C31712R (common mutation)							
Afghan patient B							
B: II-1	F/30	22	Difficulty climbing stairs	Bilateral left > right calf hypertrophy Proximal and distal LL and UL, left worse than right Waddling and steppage gait	FVC 30%, nocturnal NIV	1.5 × UNL	CBs
Italian patient C							
C: II-1	M/58	40	Distal weakness toe-walking	Prox UL, prox and dist LL	Yes	3 × UNL	Necrotic fibers, fibrosis
Italian patient D							
D: II-1	M/56	47	Myalgia, steppage gait	Severe neck flexors, severe distal UL and LL, mild-to-moderate proximal LL, mild proximal UL	Mild	2–3 × UNL	CBs, RV
Spanish patient E							
E: II-1	F/59	50	Distal weakness, respiratory failure	Proximal and distal weakness, finger extension	FVC 30%, NIV	Normal	Myopathy
Russian family F							
F: I-2	F/78^a^	58	Distal weakness	Steppage gait, mild proximal LL and finger extension	N/A	N/A	N/A
F: II-1	M/41^a^	38	Distal weakness	Mild tibialis anterior	N/A	N/A	N/A
F: II-3	M/70^a^	47	Steppage gait	Severe distal LL (tibialis anterior), mild UL (finger extension)	N/A	N/A	N/A
F:III-2	M/59	30	Ankle dorsiflexion weakness	Severe distal LL (tibialis anterior 1/5), asymmetric UL (finger extension 2/5, 3/5), mild neck flexors and proximal LL	FVC 49%	1.5 × UNL	N/A
F:IV-1	F/30	30	N/A	Tibialis anterior (4/5), finger extension (4/5)	N/A	N/A	N/A
TTN A-band-exon 344 c.95187G>C p.W31729C (re-occurring mutation)							
Portuguese patient G							
G: II-1	M/71	55	Steppage gait	Distal LL, mild kyphosis, scapular winging	Severe, invasive ventilation	Normal	CBs
Portuguese patient H							
H: II-1	M/62	55	Left foot drop	Severe distal LL, mild proximal LL and UL (4)	VC 49% nocturnal NIV	N/A	Myopathy
TTN A-band-exon 344 c.95351C>T p.A31784V (novel mutation)							
French family I							
I:III-1	F/70	55	Dyspnea	Pelvic girdle, neck flexors, ankle dorsiflexion, dysphonia	FVC 30%, nocturnal NIV	1.5 × UNL	N/A
I:III-5	F/73	24	Pelvic girdle weakness	Hip flexion, neck flexors abdominal muscles, scapular winging, ankle dorsiflexion, finger extensors	FVC 29%, nocturnal NIV	*N*	Myofibrillar aggregates
I:III-6	F/67	54	Respiratory failure	Hip flexion, neck flexors, trunk muscles, dysphonia	FVC 42%, nocturnal NIV	*N*	N/A
French family J							
J: I-1	M/50^a^	N/A	N/A	Distal LL	Yes	N/A	CBs
J: II-5	F/58^a^	44	Distal LL weakness	Proximal and distal LL, proximal UL, axial weakness, scapular winging	Yes, FVC 70%	1.5 × UNL	CBs
Argentinian patient K							
K: II-1	M/54	40	Dyspnea	Deltoid (4/5), iliopsoas and quadriceps (4/5), tibialis anterior (4−/5, 4/5), toe extension (4−/5, 4/5), unable to walk on heels, steppage gait, calf hypertrophy	FVC 45%, NIV	2.5 × UNL	Normal
TTN A-band-exon 344 c.95358C>G p.N31786K (re-occurring mutation)							
Iranian patient L							
L: II-7	M/42^a^	26	Difficulty climbing stairs	Generalized muscle weakness and atrophy	Invasive ventilator	2 × UNL	CBs

*CBs* cytoplasmic bodies, *CK* creatine kinase, *F* female, *FEV1* forced expiratory volume in one second, *FVC* forced vital capacity, *LL* lower limbs, *M* male, *MRC* Medical Research Council Scale, *N/A* not available, *NIV* non-invasive ventilation support, *RV* rimmed vacuoles, *UL* upper limbs, *UNL* upper normal limit, *VC* vital capacity, *WCB* wheelchair bound

^a^Age at death

All patients had been clinically examined by the treating neurologist, and data on nerve conduction studies and needle electromyography (EMG), creatine kinase (CK) levels in serum and muscle MRI or CT of the lower limbs were also collected. Spirometry test results were available in nine patients. An echocardiogram was performed in six patients. The diagnosis of HMERF was based on clinical symptoms of respiratory insufficiency with muscle weakness and the presence of cytoplasmic bodies in muscle biopsy, and/or on a typical pattern of muscle involvement on muscle imaging as described previously, i.e., obturator externus, semitendinosus and anterolateral muscles in the distal leg [4, 5, 8, 9, 11].

Muscle samples from the patients were snap frozen, and 8–10 µm sections were cut and examined using standard histochemical stainings. Samples were also immunostained for different myogenic antigens including myosin heavy chain isoforms (fetal, neonatal, slow and fast MyHC, MHC class I).

### Genetic studies

Genomic DNA was extracted from blood by standard methods. Direct Sanger sequencing of the titin exon 344 was performed at Emory Genetics Laboratory (http://geneticslab.emory.edu/) in patient B and Tampere Neuromuscular Research Center, Finland in patient G. Targeted next-generation sequencing (NGS) was performed as previously described [17] in patients A, C, D, and H–L. Version 2 of the MYOcap gene panel was used that is targeted to the exons of 236 genes including all known genes for muscular dystrophy or myopathy at the time. In Patient E, an NGS panel targeted to the exons of 119 genes known to cause muscular dystrophy or myopathy was performed at the genetic service of Hospital de la Santa Creu i Sant Pau, Barcelona, Spain. Targeted sequencing was ordered for the proband of Family F from a commercial laboratory (Genomed Ltd., Moscow, the list of targeted genes: http://price.genomed.ru/?testid=826).

*TTN* variants are described according to the coding DNA reference sequence (NG_011618.3 or LRG_391), covering transcript variant-IC (NM_001267550.1).

## Results

### Molecular genetic findings

#### Novel mutations

A novel A-band mutation c.95126C>A p.P31709H was identified in the proband of family A (III-3). The proband’s mother and two brothers were similarly affected with respiratory insufficiency and muscle weakness but already deceased before the study. Her affected half-sister needed mechanical ventilation at age 40.

Two French families I and J, as well as one Argentinian patient K were identified with a novel mutation c.95351C>T p.A31784V. In family I three affected patients were studied. The proband’s affected father (I-1) in family J was already deceased and not available for the study. However, the son (III-1) was genetically studied as he has mild rigid spine although no muscle involvement. He did not carry the titin mutation. The proband (II-5) and her sister (II-6) also had joint contractures and rigid spine that have probably another genetic background.

The previously unknown variants are not listed in gnomAD (v2.1), but they are listed in ClinVar as variants of uncertain significance (ClinVar ID is 283245 for c.95126C>A p.P31709H and 497143 for c.95351C>T p.A31784V). Moreover, the variant p.P31709H is also reported in dbSNP (rs869320739).

### Common mutation

Single patients B–E were found to harbor the most frequently identified *TTN* A-band mutation, c.95134T>C p.C31712R [11, 12]. No other family members of these patients were available for genetic studies. In addition, the proband of family F with several affected members carried the same mutation.

#### Re-occurring mutations

Patients G and H were identified with c.95187G>C p.W31729C previously reported in two single patients [9, 16]. In patient L the mutation c.95358C>G p.N31786K, which has also been found in one British patient, was identified [15]. DNA samples were available in five healthy family members of patient L (mother, four siblings), but none of them carried the mutation. The patient and family members were also haplotyped, and the mutation was found to be de novo in the patient as the same haplotype but not the mutation was identified in healthy family members.

### Clinical findings

Detailed clinical data are presented in Table 2. The mean age at symptom onset was 42 years (range 22–58 years). The main presenting symptoms were related to lower limb proximal or distal weakness in all patients, and to respiratory failure at onset in only two patients.

#### Clinical characteristics of patient A with the novel TTN mutation c.95126C>A p.P31709H

**Fig. 2:**
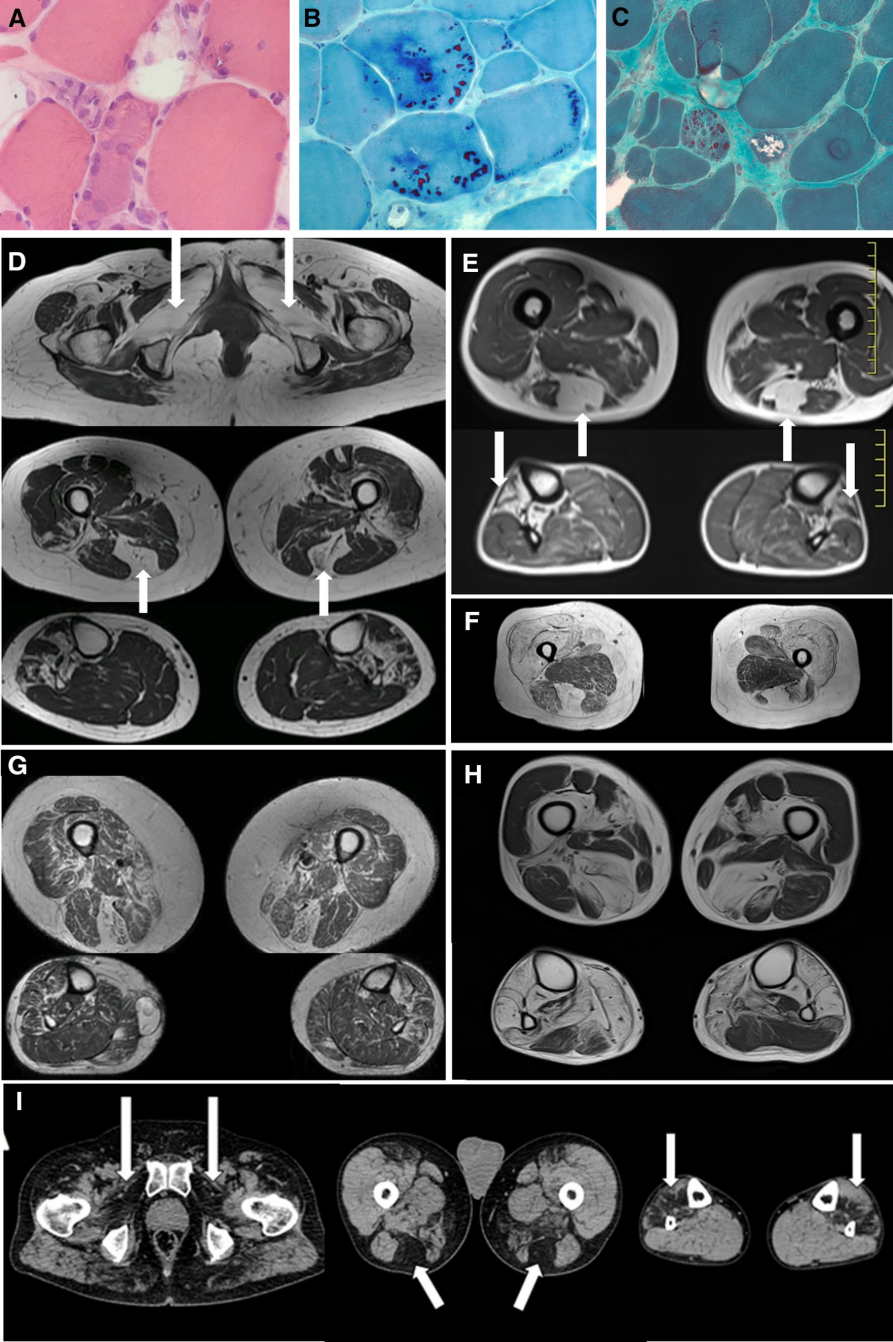
Histological and muscle imaging findings. Patient **a** haematoxylin and eosin staining shows atrophic fibers and rimmed vacuolar pathology. **b** There are numerous mostly subsarcolemmal cytoplasmic bodies (CBs) present in the biopsy from patient L with Gomori trichrome staining but CBs can be present in only occasional fibers as seen in figure **c** (patient G). **d** Muscle MRI from Family I (III-4) with the novel mutation shows typical fatty degenerative changes in obturatorius, semitendinosus and anterior lower leg muscles. The same but more severe and diffuse involvement is present in her sister (III-5) (G). The mildest form of involvement is demonstrated in E (patient E) and more advanced fatty degeneration in F (patient B) and H (family F III-2). CT images in figure I (patient K) show the most typical changes in HMERF marked with arrows

A 52-year-old female presented with slowly progressive proximo-distal myopathy starting at age 31 years. She had respiratory insufficiency and a need for a non-invasive ventilation (NIV). Muscle biopsy at age 45 years showed rimmed vacuoles and subtle lesions compatible with myofibrillar aggregations (Fig. 2a). No cytoplasmic bodies were observed. Muscle imaging could not be performed due to patient’s claustrophobia.

#### Clinical characteristics of families I–K with the novel c.95351C>T p.A31784V mutation

All three patients in family I were followed up for severe respiratory failure which progressed slowly since adolescence. One of the patients had been diagnosed with asymptomatic restrictive reduced respiratory capacity after a systematic screening by the school doctor at the age of 12. However, respiratory symptoms, i.e., dyspnea did not become apparent before the age of 50 years in the patient, which resulted in the need for non-invasive nocturnal ventilation. Further, patient II-3 had died at the age of 51 years due to respiratory failure after general anesthesia and was diagnosed with pulmonary embolism at that time. Muscle weakness in the family was predominant at the pelvic girdle, i.e., hip flexion, but also neck flexors and trunk muscles were weak. Ankle dorsiflexion and finger extension strength was less severely affected. One patient presented with more severe weakness including limitation of arm abduction. She needed bilateral help for walking. There was no calf hypertrophy or facial weakness, but dysphonia was noted in patients III-1 and III-6. They all had kyphoscoliosis since adolescence. Two patients underwent echocardiography with normal results. Myofibrillar aggregates were present in the muscle biopsy.

The proband (II-5) and her father in Family J showed onset of symptoms in the distal lower limbs. Respiratory insufficiency was also present in both patients. In addition to rigid spine, mandibular and ankle contractures, there were axial weakness and scapular winging in the proband and she needed a stick for walking. Muscle biopsies in both showed cytoplasmic bodies compatible with HMERF findings.

Patient K is a 54-year-old male with severe respiratory insufficiency, proximal weakness, and no family history. At the age of 40 the first respiratory symptoms and decreased vital capacity (58%) were noted. Muscle symptoms appeared after a few years with proximal upper and lower limb weakness. He was also unable to walk on heels. Respiratory insufficiency progressed to the vital capacity of 45% at age 54 years. EMG was myopathic and serum CK level was elevated, but no abnormal findings were observed in the muscle biopsy (vastus lateralis at age 45 years). Cardiac examination including echocardiogram was also normal. His mother suffered from dyspnea and died suddenly in her sleep at age 70 years.

#### Patients B–F with the common TTN mutation c.95134T>C p.C31712R

The presenting symptoms of the patients were distal lower leg weakness usually starting with ankle dorsiflexion weakness and respiratory insufficiency. The age of onset varied from 20 to 50 years of age. Muscle weakness slowly progressed to encompass both upper and lower limb muscles proximally and distally. Neck flexor and finger extensor weakness were common findings. Respiratory symptoms ranged from asymptomatic or mild to severe with a need for NIV. CK levels were normal or mildly elevated. Muscle biopsy findings were available from four families and revealed cytoplasmic bodies and/or unspecific myopathic/dystrophic changes (Fig. 2b, c).

In retrospect, family history of these patients was positive although none of the relatives had been diagnosed with a specific muscle disease. Patient B had a 41-year-old brother who had similar symptoms since age 32 and used NIV 24 h a day. The mother of patient D who had died at the age of 67 years due to heart attack, had gait difficulties since aged 40 followed by respiratory insufficiency, while patient E had a sister who had muscle weakness and died because of sudden death around 50 years of age. In addition to the proband and his daughter in Family F, the proband’s father, paternal uncle and grandfather had similar symptoms.

#### Clinical characteristics of patients G, H, and L with re-occurring TTN mutations

Patient G, a 71-year-old male patient presented with distal weakness in the lower limbs starting at the age of 55. He presented steppage gait, mild kyphoscoliosis and scapular winging. He also had severe respiratory involvement (since the age of 68) and needed continuous mechanical ventilation. No signs of cardiomyopathy were revealed by echocardiogram. Muscle biopsy showed cytoplasmic bodies. He had no clear family history although his father, who died at age 68 years, had severe muscle weakness, and the father’s sister was wheelchair-bound without a known cause.

Patient H is a 62-year-old male whose symptoms began at age 55 with foot drop, first on the left, then on the right side. He had indications of nocturnal hypoxemia, and respiratory evaluations showed reduced VC (sitting 48.9%, prone 32.3%) necessitating nocturnal NIV. In addition, mild proximal weakness in the upper and lower limbs was noted. EMG was myopathic, more severe in the anterior leg muscles. Muscle histology revealed only unspecific myopathic changes. The proband had ten brothers of which seven were already deceased. Several siblings had gait disturbances and some cognitive deterioration but were not available for evaluation. The father died at 74 years due to cardiac disease and had similar steppage gait but no cognitive impairment.

Patient L is a 40-year-old male with difficulty in climbing stairs starting aged 26 and no family history. On the first examination, proximal muscle weakness in addition to steppage gait, calf hypertrophy, and macroglossia were present. The weakness was prominent in the shoulder and pelvic girdle, finger extensors and rhomboid muscles; facial muscles were spared. Respiratory failure developed at the age of 30, and at the last examination at age 40 he was bedridden and in need of mechanical ventilation. Echocardiography revealed a mildly decreased ejection fraction of 50% and mild dilated cardiomyopathy. Cytoplasmic bodies were present in his muscle biopsy. The patient died at age 42 as a result of respiratory failure.

#### Muscle imaging

Muscle MRI was performed in ten patients and CT in two patients. The most typical finding was fatty degenerative changes in semitendinosus and anterolateral muscles of the distal lower leg. In addition, at the pelvic level iliopsoas and gluteal muscles were affected and at the thigh level quadriceps and gracilis in some of the patients (Fig. 2d–i).

## Discussion

Our study on 12 HMERF families shows that the disease is not that rare as previously understood and can be found worldwide. Together with two novel mutations there are more than ten different *TTN* A-band mutations identified leading to typical muscle imaging and histology findings [4, 5, 11]. Although the mutations reported here are dominant many of our patients were single cases rendering the diagnosis of a dominant disease challenging.

Muscle weakness in HMERF together with early respiratory failure usually involves proximal, distal and trunk muscles as the disease progresses. In contrast to most muscular dystrophies the majority of reported HMERF patients either had respiratory insufficiency among the presenting symptoms or developed failure later during the disease course (Table 1). In our study, the mean age of onset of respiratory symptoms was 50.3 years (range 30–68 years). Three patterns of presenting symptoms can be delineated: (1) distal myopathy especially with ankle dorsiflexion weakness; (2) pelvic girdle weakness, or (3) respiratory insufficiency as a first sign, with the distal presentation being most common in our patients (9/19). Despite variable distribution of muscle symptoms, most frequently reported affected muscles were neck flexors, finger extensors, ankle dorsiflexors and proximal lower limb muscle weakness (Table 1), which are affected also in many of our patients [4, 5, 9]. Less frequently reported features such as kyphosis, scapular winging, dysphonia or calf hypertrophy [18] were rarely present. Coexisting cardiac symptoms, i.e., arrhythmias were found in HMERF patients with the common c.95134T>C p.C31712R mutation [19]. Only one of our patients had signs of mild dilated cardiomyopathy (patient L), and cardiac abnormalities occurred very rarely also in the previous reports [4, 5, 9, 18].

The hallmarks of the disease, i.e., cytoplasmic bodies in muscle biopsy and typical distribution of muscle involvement on imaging, i.e., semitendinosus and anterolateral muscles in the distal leg, were frequent findings also with the novel mutations. Cytoplasmic bodies, rimmed vacuoles and/or myofibrillar aggregates were seen in eight out of 12 patients studied. Thus typical diagnostic changes were not detected in all; however, immunohistochemical stainings, e.g., myotilin, desmin or p62, to improve detection of cytoplasmic bodies or myofibrillar aggregates [15] were not consistently used in the patients with consistent diagnosis. Further, diagnostic findings can be missed in routine examinations as cytoplasmic bodies and rimmed vacuoles can be present only in rare fibers, which apparently is one reason for difficulties to reach a correct diagnosis.

Several dominant mutations in titin A-band have been identified in HMERF patents, c.95134T>C p.C31712R being the most frequent. The common mutation was now diagnosed in patients from the Middle East, South America and Russia. This shows that it is not restricted to Europe, one haplotype or founder mechanism as previously thought [15, 20]. We expand the mutational spectrum with two novel mutations identified in four families resulting in typical generalized muscle weakness and respiratory symptoms. In addition to the common mutation, the other reported mutations have been identified in single patients [11, 12]; two of them now found in our patients.

## References

[1] Chapon F, Viader F, Fardeau M (1989). Familial myopathy with “cytoplasmic body” (or “spheroid”) type inclusions, disclosed by respiratory insufficiency (in French). Rev Neurol (Paris).

[2] Edström L, Thornell LE, Albo J, Landin S, Samuelsson M (1990). Myopathy with respiratory failure and typical myofibrillar lesions. J Neurol Sci.

[3] Chinnery PF, Johnson MA, Walls TJ (2001). A novel autosomal dominant distal myopathy with early respiratory failure: clinico-pathologic characteristics and exclusion of linkage to candidate genetic loci. Ann Neurol.

[4] Ohlsson M, Hedberg C, Brådvik B (2012). Hereditary myopathy with early respiratory failure associated with a mutation in A-band titin. Brain.

[5] Pfeffer G, Elliott HR, Griffin H (2012). Titin mutation segregates with hereditary myopathy with early respiratory failure. Brain.

[6] Pfeffer G, Povitz M, Gibson GJ, Chinnery PF (2015). Diagnosis of muscle diseases presenting with early respiratory failure. J Neurol.

[7] Naddaf E, Milone M (2017). Hereditary myopathies with early respiratory insufficiency in adults. Muscle Nerve.

[8] Birchall D, von der Hagen M, Bates D, Bushby KM, Chinnery PF (2005). Subclinical semitendinosus and obturator externus involvement defines an autosomal dominant myopathy with early respiratory failure. Neuromuscul Disord.

[9] Palmio J, Evilä A, Chapon F (2014). Hereditary myopathy with early respiratory failure: occurrence in various populations. J Neurol Neurosurg Psychiatry.

[10] Izumi R, Niihori T, Aoki Y (2013). Exome sequencing identifies a novel TTN mutation in a family with hereditary myopathy with early respiratory failure. J Hum Genet.

[11] Uruha A, Hayashi YK, Oya Y (2015). Necklace cytoplasmic bodies in hereditary myopathy with early respiratory failure. J Neurol Neurosurg Psychiatry.

[12] Savarese M, Sarparanta J, Vihola A, Udd B, Hackman P (2016). Increasing role of titin mutations in neuromuscular disorders. J Neuromuscul Dis.

[13] Yue D, Gao M, Zhu W (2015). New disease allele and de novo mutation indicate mutational vulnerability of titin exon 343 in hereditary myopathy with early respiratory failure. Neuromuscul Disord.

[14] Toro C, Olivé M, Dalakas MC (2013). Exome sequencing identifies titin mutations causing hereditary myopathy with early respiratory failure (HMERF) in families of diverse ethnic origins. BMC Neurol.

[15] Pfeffer G, Barresi R, Wilson IJ (2014). Titin founder mutation is a common cause of myofibrillar myopathy with early respiratory failure. J Neurol Neurosurg Psychiatry.

[16] Bugiardini E, Morrow JM, Shah S (2018). The Diagnostic Value of MRI Pattern Recognition in Distal Myopathies. Front Neurol.

[17] Evilä A, Arumilli M, Udd B, Hackman P (2016). Targeted next-generation sequencing assay for detection of mutations in primary myopathies. Neuromuscul Disord.

[18] Tasca G, Udd B (2018). Hereditary myopathy with early respiratory failure (HMERF): still rare, but common enough. Neuromuscul Disord.

[19] Steele HE, Harris E, Barresi R (2016). Cardiac involvement in hereditary myopathy with early respiratory failure: a cohort study. Neurology.

[20] Pfeffer G, Sambuughin N, Olivé M, Tyndel F, Toro C, Goldfarb LG, Chinnery PF (2014). A new disease allele for the p.C30071R mutation in titin causing hereditary myopathy with early respiratory failure. Neuromuscul Disord.

